# Dissecting the causal links between gut microbiome, immune traits and polyp using genetic evidence

**DOI:** 10.3389/fimmu.2024.1431990

**Published:** 2024-09-13

**Authors:** Cheng Zhou, Xiaofeng Ye, Zhinuo Liu, Tong Liu, Shanzheng Li, Jinqiu Yang, Jingjing Wei, Peng Yu, Ran Jia, Wenxia Zhao

**Affiliations:** ^1^ Department of Gastroenterology, Changzhou Hospital of Traditional Chinese Medicine Affiliated to Nanjing University of Chinese Medicine, Changzhou, China; ^2^ The First College of Clinical Medicine, Henan University of Chinese Medicine, Zhengzhou, China; ^3^ Heart Center, The First Affiliated Hospital of Henan University of Chinese Medicine, Zhengzhou, China; ^4^ Department of Gastroenterology, The First Affiliated Hospital of Henan University of Chinese Medicine, Zhengzhou, China

**Keywords:** Mendelian randomization, gut microbiota, immune cell, nasal polyp, gastric polyp, colon polyp

## Abstract

**Background:**

Previous research has demonstrated an association between gut microbiota and immune status with the development of several diseases. However, whether these factors contribute to polyps remains unclear. This study aims to use Mendelian randomization (MR) to investigate the causal relationship between gut microbiota and 4 types of polyps (nasal, gallbladder, colon, and gastric polyps), as well as to analyze the mediating role of immune traits.

**Methods:**

This study utilized large-scale GWAS meta-analyses of gut microbiota (MiBioGen Consortium), 731 immune traits, and 4 types of polyps (one from the FinnGen Consortium and three from the NBDC Human Database). Univariate MR with the inverse variance weighted (IVW) estimation method was employed as the primary analytical approach. A two-step MR analysis was performed to identify potential mediating immune traits. Additionally, multivariable MR approach based on Bayesian model averaging (MR-BMA) was employed to further prioritize gut microbiota and immune traits associated with polyp development.

**Results:**

Based on IVW method in univariate MR analysis, we identified 39 gut microbial taxa and 135 immune traits significantly causally associated with at least one type of polyp. For nasal polyps, 13 microbial taxa and 61 immune traits were causally associated. After false discovery rate (FDR) correction, CD3 on Central Memory CD8^+^ T cells and CD3 on CD4 regulatory T cells remained significant. MR-BMA identified 4 gut microbial taxa and 4 immune traits as high priority. For gallbladder polyps, 9 microbial taxa and 30 immune traits were causally associated. MR-BMA identified 8 microbial taxa and 6 immune traits as higher importance. For colon polyps, 6 microbial taxa and 21 immune traits were causally associated. MR-BMA identified 4 microbial taxa and 3 immune traits as higher importance. For gastric polyps, 12 microbial taxa and 33 immune traits were causally associated. *Actinobacteria* remained significant after FDR correction, and MR-BMA identified 7 gut microbial taxa and 6 immune traits as high priority. We identified 16 causal pathways with mediator directions consistent with the direction of gut microbiome-polyp association. Of these, 6 pathways were associated with the mechanism of nasal polyps, 1 with gallbladder polyps, 2 with colon polyps, and 7 with gastric polyps.

**Conclusions:**

Our findings shed light on the causal relationships between gut microbiota, immune traits, and polyp development, underscoring the crucial roles of gut microbiota and immune status in polypogenesis. Furthermore, these findings suggest potential applications in polyp prevention, early screening, and the development of effective strategies to reduce polyp risk.

## 1 Introduction

Growths on the mucosal surfaces of the human body are collectively referred to as polyps, encompassing various pathological conditions such as hyperplasia, inflammation, and adenomas. While polyps are primarily classified according to their site of occurrence, including nasal polyps, gallbladder polyps, colon polyps, and gastric polyps, among others, this anatomical categorization is merely a starting point in the multidimensional process of polyp classification. In clinical practice and research, more refined and standardized classification systems are employed, considering not only the anatomical location but also morphological characteristics, size, surface features, histological type, and potential malignancy risk. These specialized systems, such as the NICE (NBI International Colorectal Endoscopic) classification for colon polyps, the Paris Classification for gastrointestinal polyps, and various methods for gallbladder and nasal polyps, provide crucial guidance for clinical decision-making and treatment strategies. While most polyps are benign, a small proportion can be malignant, underscoring the importance of accurate classification. Among individuals undergoing gastroscopy, the incidence of gastric polyps is 6.35%, with 77% being fundic gland polyps, 17% hyperplastic polyps, and malignant tumors being relatively uncommon (1). Polyps often develop insidiously, without specific clinical symptoms, and their pathogenesis remains unclear. Polyp development is closely associated with endocrine disorders, genetics, age, medications, and lifestyle habits (2). Notably, immune imbalance due to gut microbiota dysbiosis is considered a potential risk factor for polyp formation.

Microorganisms and the host body maintain a balanced relationship, with most microorganisms participating in host metabolism. These microorganisms aid digestion, promote vitamin synthesis, stimulate the development of the intestinal immune system, and together with epithelial cells, form an intrinsic barrier against pathogen invasion. Numerous studies have demonstrated that the fecal microbial composition of individuals with colon polyps significantly differs from that of healthy individuals (3, 4). Furthermore, early immunological changes associated with polyps may be linked to the microenvironment resulting from gut microbiota dysbiosis. Notably, bacterial drug delivery systems targeting the gut microbiota may emerge as an important therapeutic approach for both colon polyps and colorectal cancer (5). Since the late 20th century, *Helicobacter pylori* has been recognized as a crucial microorganism in the development of gastric polyps (6). Following numerous clinical studies validating its role, the eradication of *H. pylori* has become a routine treatment for gastric polyps (7). The gallbladder is directly connected to the intestine, with the bile acid cycle serving as an important link between these two organs. Hu et al. (8) demonstrated that intestinal microbiota, especially *Desulfovibrionales*, can induce gallbladder disease by increasing the secretion of secondary bile acids and enhancing bile acid hydrophobicity. For non-intestinal diseases, the intestinal microbiota and their metabolites can affect distant organs through the bloodstream or lymphatic circulation, either by active absorption or disruption of the epithelial barrier (9, 10). Therefore, the role of the intestinal microbiota should not be disregarded in the development of polyps, including nasal polyps, gallbladder polyps, colon polyps, and gastric polyps. Metabolic abnormalities resulting from gut microbial dysbiosis have been identified as a risk factor for chronic sinusitis (11). Chronic sinusitis frequently coexists with nasal polyps, characterized by eosinophil recruitment due to abnormal activation of immune cells, such as CD4^+^ T cells and Th2 cells (12). Notably, this localized immune remodeling, in turn, is considered an important contributor to nasal polyp development (13). Therefore, we focused on analyzing the intestinal microbiota and 731 immune profiles to investigate their potential associations with polyp development.

The clinical diagnosis of polyps relies primarily on endoscopy, including gastroenteroscopy and capsule endoscopy. These endoscopic examinations can be complemented by non-invasive imaging techniques (ultrasound and magnetic resonance imaging) to evaluate the size and type of polyps. Endoscopic resection techniques, including strangulation polypectomy, endoscopic mucosal resection, and endoscopic submucosal dissection, provide the most direct and effective means for diagnosing and treating polyps. However, these invasive procedures may not be suitable for all patients. For certain patients, treatment involves symptomatic and etiologic interventions, coupled with regular follow-up monitoring to assess polyp size progression. However, for the majority of patients, polyps tend to grow, necessitating eventual endoscopic surgery to halt disease progression and prevent malignant transformation. Therefore, identifying polyp-related biomarkers could provide new insights into the pathogenesis of polyps and contribute to polyp prevention, early screening, and the development of effective strategies to mitigate polyp risk.

Mendelian randomization (MR) is a method used to evaluate the causal relationship between an exposure and an outcome, utilizing single nucleotide polymorphisms (SNPs) strongly associated with the exposure as instrumental variables (IVs) (14). This approach is based on Mendelian laws of inheritance, treating genotypes as naturally randomized factors. MR helps mitigate the effects of reverse causation and confounding factors, providing more reliable causal inferences. By studying the human genome, genetic variants associated with certain diseases can be identified, shedding light on disease pathogenesis and providing a basis for diagnosis and treatment. In this study, we employed a two-sample bidirectional and mediated MR design to assess the causal relationship between gut microbiota and 4 types of polyps (nasal, gallbladder, colon, and gastric polyps), as well as to investigate the potential mediating role of immune profiles. Additionally, multivariable MR approach based on Bayesian model averaging (MR-BMA) was employed to identify the most likely gut microbiota and immune traits associated with polyp development.

## 2 Materials and methods

### 2.1 Study design

**Figure 1:**
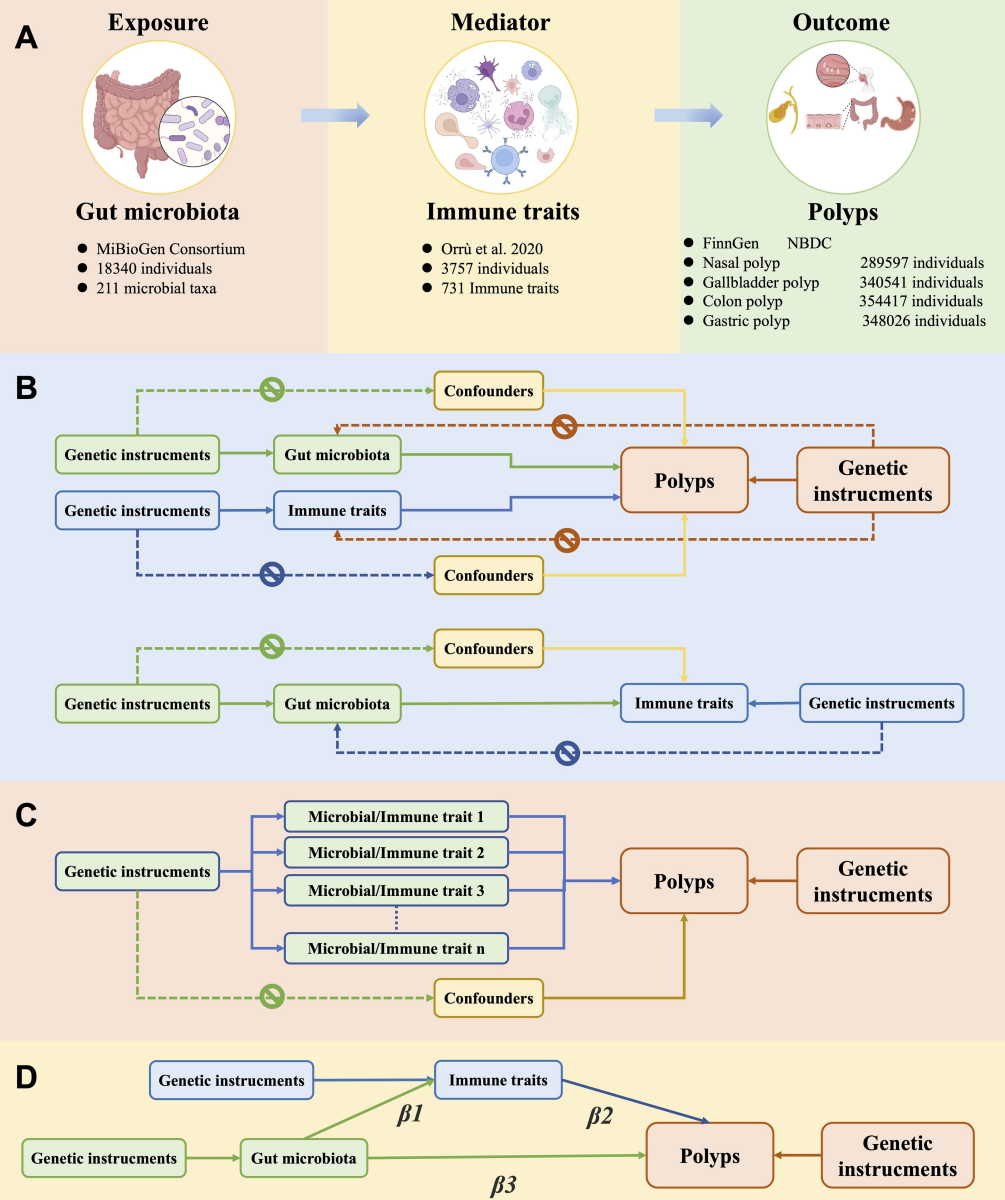
Overview of the study design and methods. **(A)** Details on exposure, mediator, and outcome data. **(B)** Univariate Mendelian randomization (MR) analyses with three components: 1) exposure to gut microbes and polyp as outcome, 2) exposure to immune traits and polyp as outcome, 3) exposure to gut microbiota and immune traits as outcome. Genetic instrumental variables were associated solely with exposure factors, did not directly influence polyp outcomes independently of these exposures, and were unaffected by confounding factors. **(C)** Multivariate MR analyses that ranked the importance of exposure factors using MR-BMA of positive exposure outcomes. **(D)** Mediating MR analysis to assess the mediating role of immune traits in the causal relationship between gut microbiota and polyps.

Univariate Mendelian randomization (MR) analysis was employed to investigate the causal relationship between gut microbiota, immune traits, and 4 types of polyps in human (nasal, gallbladder, colon, and gastric polyps). A two-step MR approach was performed to assess the potential mediating effects of immune traits. Additionally, multivariate MR analysis based on Bayesian model averaging (MR-BMA) was employed to rank the importance of gut microbiota and immune traits. Single nucleotide polymorphisms (SNPs) associated with these factors were utilized as instrumental variables (IVs). The MR study was based on the following three assumptions (15): (1) SNPs are strongly associated with the exposure factors; (2) SNPs are independent of potential confounding factors; (3) SNPs influence the outcome only through the exposure factors. See **Figure 1** for the study flowchart.

### 2.2 Source of GWAS summary statistics

GWAS data on gut microbiota were obtained from the MiBioGen consortium (https://mibiogen.gcc.rug.nl), comprising a total of 18,340 participants across 24 cohorts, including 13,266 participants from 16 European cohorts (16). The gut microbiota data encompassed a total of 211 taxa, including 131 genera, 35 family, 20 order, 16 class, and 9 phyla. GWAS data for 731 immune traits were derived from a cohort of 3,757 Sardinian individuals (17). The 731 immune traits could be categorized into 7 immune cell types: B cells (n=190), dendritic cells (n=64), T cell maturation stages (n=79), monocytes (n=43), myeloid cells (n=64), TBNK cells (n=124), and Treg cells (n=167). Data on polyps were obtained from two large databases. GWAS data (version R9) for nasal polyps, comprising 6,255 cases and 283,342 controls, were obtained from the FinnGen consortium (https://www.finngen.fi/fi) (18). The FinnGen research project is a nationwide Finnish genetic study that comprehensively collects genetic and electronic health record data, providing an important resource for studying disease-associated genetic variants. Data on gastric polyps (6,155 cases and 341,871 controls), colon polyps (22,049 cases and 332,368 controls), and gallbladder polyps (458 cases and 340,083 controls) were obtained from the NBDC Human Database (https://humandbs.biosciencedbc.jp/en/), a platform established by the NBDC Program Department of the Japan Science and Technology Agency (JST) for sharing various human-related data. The data for these three polyp types were derived from a European population study that included genome-wide data for 628,000 European individuals, with most of the data originating from the FinnGen consortium and the UK Biobank (19).

### 2.3 Selection of genetic variants

Independent SNPs form the basis of MR studies. Consistent with previous research (20), we selected SNPs with a p-value < 1 × 10^−5^ as instrumental variables. To exclude SNPs in linkage disequilibrium, we set k to 500 kb and r^2^ to 0.1 (21). To mitigate the risk of bias due to weak instrumental variables, we calculated the general F-statistic for each exposure factor. The F-statistics for SNPs associated with gut microbiota ranged from 14.59 to 88.43 (**Supplementary File S1**), while those for immune traits ranged from 19.55 to 2381.77 (**Supplementary File S2**), indicating strong correlations (F-statistic > 10) (22). To avoid the influence of confounding factors, we also removed SNPs strongly associated with alcohol consumption and smoking. Regarding potential sample overlap between exposure factors and outcomes, we consulted the relevant literature and found no sample overlap in the GWAS data used for exposures and outcomes.

### 2.4 Univariate Mendelian randomization

Analyses were conducted using the TwoSampleMR, MRPRESSO, and MendelianRandomization packages in R Studio (version 4.3.2) (23). In preliminary analyses, when the number of SNPs ≥2, we employed the random-effects inverse variance weighted (IVW) method, which is widely recognized as the most robust approach in MR analyses, to estimate the potential effect of exposure factors on polyp development. Additionally, we utilized the MR Egger, Weighted Median, Simple mode, and Weighted mode methods for the analysis (24–26). A heterogeneity test was employed to assess differences between IVs, and Cochran’s Q test was used to evaluate heterogeneity among IVs. The MR-Egger intercept and MR-PRESSO methods were utilized to detect horizontal pleiotropy (27, 28). Horizontal pleiotropy is when the factors we use to explore a link between two things might be affecting the outcome in more than one way, not just through the pathway we’re looking at. MR-Egger regression is a method that handles this by using a special kind of regression analysis that includes an extra term, the intercept. This intercept helps us understand how much the factors might be affecting the outcome in ways we didn’t expect. The slope of the regression line gives us a fair estimate of the actual effect we’re interested in. MR-PRESSO is a way to check if these unexpected effects, or pleiotropy, are happening with all the factors we’re using in our study. It has three main steps: First, it does a global test to see if there’s any sign of these extra effects. Second, it finds any outliers, or factors that seem to be behaving very differently, and removes them to get a corrected result. Third, it compares the results before and after removing these outliers to see if there’s a big difference. If the global test’s p-value is less than 0.05, it suggests that there are indeed some unexpected effects at play. When the number of SNPs <2, we employed the Wald ratio method for sensitivity analysis. To confirm the directionality of the MR analyses, we utilized the mr_steiger function to assess whether the results indicated a causal effect on the exposure factors. The mr_steiger function evaluates the putative direction of causality by quantifying the correlation coefficients of the SNPs’ effect sizes on both the exposure and outcome, while also accounting for sample size. It yields p-values that serve as a statistical measure to infer the likelihood of a directional causal relationship from exposure to outcome.

### 2.5 Multivariable MR approach based on Bayesian model averaging

Traditional multivariate Mendelian randomization (MVMR) analysis, which relies on standard linear regression, performs suboptimally when dealing with a large number of risk factors. Multivariable MR approach based on Bayesian model averaging (MR-BMA) is a Bayesian variable selection method for identifying potential causal determinants of disease from numerous candidate risk factors. As a novel multivariate MR approach, MR-BMA is well-suited for high-throughput sequencing studies with large data volumes, as it analyzes all possible combinations of risk factors and facilitates biomarker screening. We conducted MR-BMA analyses for significant gut microbiota and immune traits. The number of repeated iterations was set to 10,000, the prior probability was set to 0.1, and the prior variance was set to 0.5. The marginal inclusion probability (MIP), defined as the sum of the posterior probabilities (PP) for all specific models (i.e., a single biomarker or a combination of biomarkers), was calculated for each exposure factor. Additionally, the model-averaged causal estimate (MACE) was calculated, representing the direct causal effect of the biomarker averaged across all relevant models on polyp development. Exposures within the same category were ranked based on their MIP, with an MIP >0.1 considered indicative of high importance. All analyses were performed in R Studio (version 4.3.2).

### 2.6 Mediation analysis

Our study employed a two-step MR approach to evaluate the potential mediating effect of immune traits on the relationship between gut microbiota and 4 types of polyps. Specifically, we sought to identify microbial taxa which displayed causal associations with the 4 types of polyps through their impacts on immunity. The causal effects of gut microbiota on polyps (Beta3), gut microbiota on immune traits (Beta1), and immune traits on polyps (Beta2) were analyzed separately using univariate MR. The proportion of the total effect mediated by the immune traits was estimated by dividing the indirect effect (Beta1×Beta2) by the total effect (Beta3). This analysis is grounded in the methodology of mediated effects analysis, aimed at elucidating influence of intestinal microbiota on the development of polyps via immune-mediated pathways. Here, β3 signifies the comprehensive effect of intestinal microbiota on polyps, encompassing both direct and indirect mechanisms; Beta1 quantifies the impact of intestinal microbiota on immune traits, whereas Beta2 captures the effect of these immune traits on polyps. By estimating the proportion of the indirect effect (Beta1×Beta2) within the total effect (Beta3), we quantitatively assess the magnitude of the mediating role played by immune traits in the intestinal microbiota-polyps relationship. This methodological approach facilitates a deeper understanding of intricate causal networks and enables the evaluation of the relative significance of various pathways of influence.

## 3 Result

### 3.1 Results of the weak instrumental variable test

The F-statistics for each SNP in gut microbiota ranged from 14.59 to 88.43 (**Supplementary File S1**), and the F-statistics for the immune traits ranged from 19.55 to 2381.77 (**Supplementary File S2**), all with high correlations (F-statistics >10).

### 3.2 Causal relationships between the gut microbiota, immune traits and nasal polyp

#### 3.2.1 Univariate MR analysis and MR-BMA analysis of gut microbiota on nasal polyp

**Figure 2:**
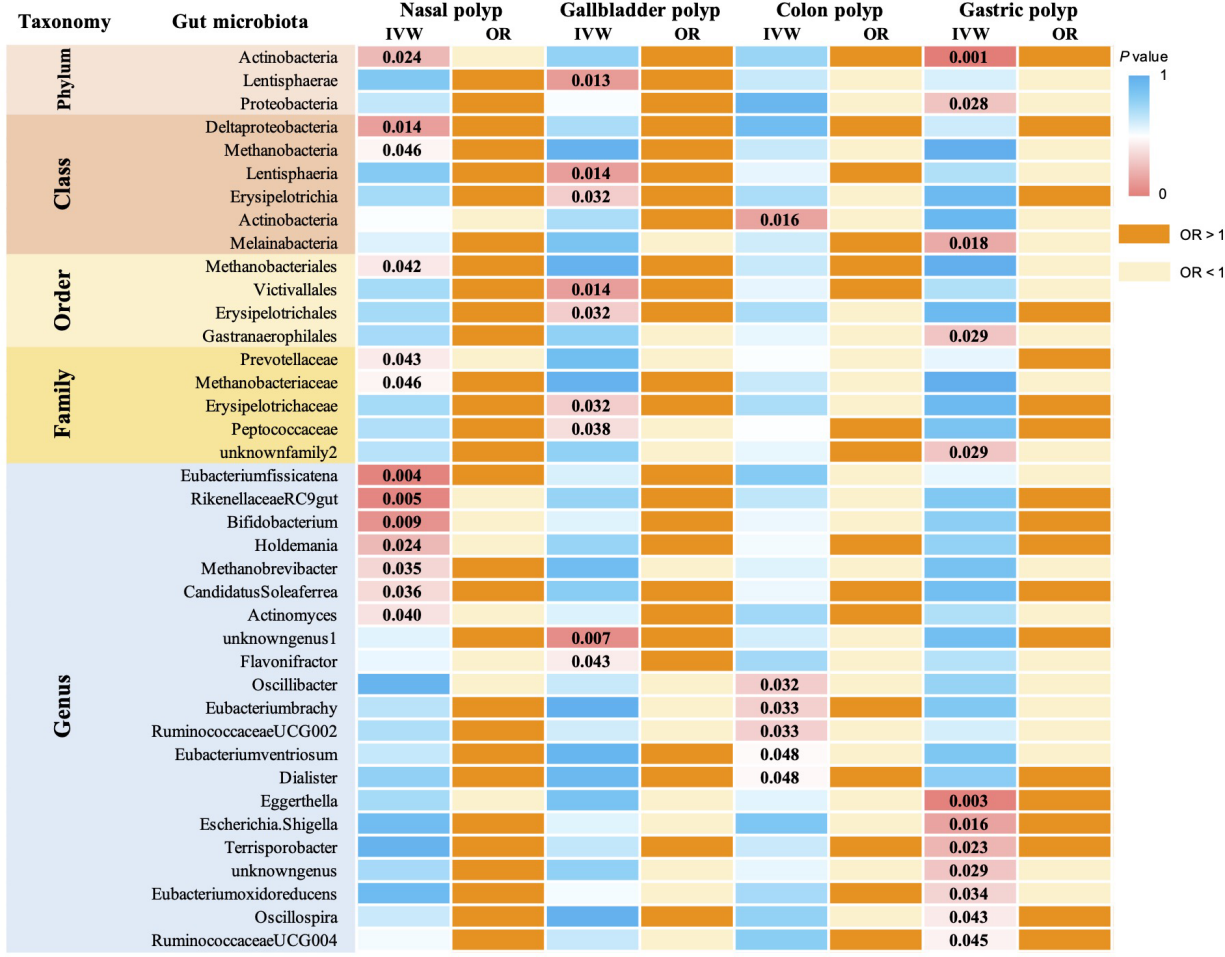
MR analysis examined the causal effect of gut microbiota on 4 types of polyps. The analysis identified 39 gut microorganisms that had a significant causal effect on at least 1 type of polyp, and their corresponding p-values are reported. Odds ratio (OR) greater than 1 indicates that the gut microorganism is a risk factor for polyps, while OR less than 1 suggests a protective effect.

**Figure 3:**
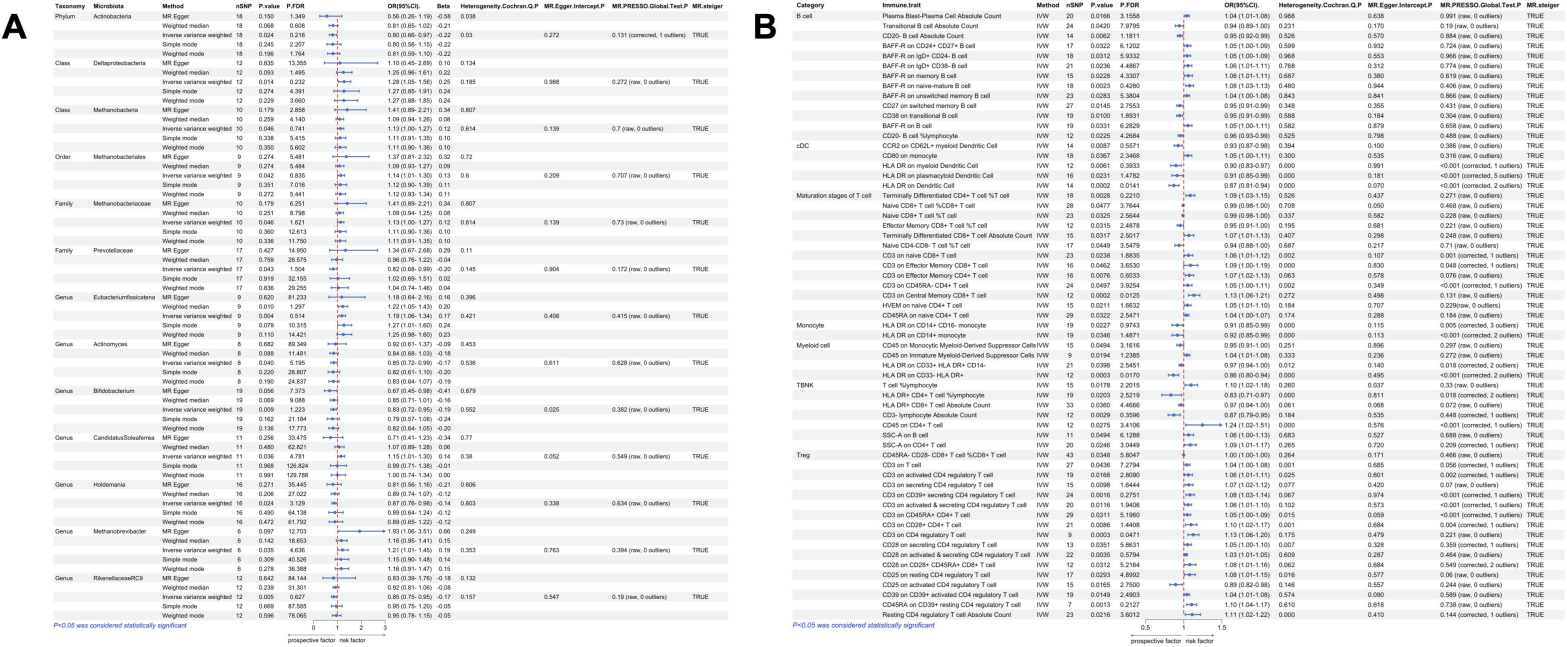
Univariate MR results of gut microbiota and immune traits on nasal polyps. **(A)** MR forest plot of the causal effect of gut microbiota on nasal polyps. **(B)** MR forest plot of the causal effect of immune traits on nasal polyps. OR>1 indicates an increased risk, whereas OR<1 indicates a decreased risk. The last 4 columns are analyses of horizontal pleiotropy, heterogeneity, and the directionality of MR, with P > 0.05 indicating no pleiotropy or heterogeneity and TRUE indicating no reverse causality.

According to the IVW method, we identified a total of 39 gut microbiota taxa that were significantly causally associated with at least one type of polyp, of which 13 microbial taxa were potentially associated with nasal polyps (**Figure 2**). In assessing the causal effect of gut microbes on nasal polyps, we identified 1phylum, 1 family, and 4 genera as potential protective factors against the development of nasal polyps. The identified taxa were: *Actinobacteria* (OR=0.80, 95%CI:0.66-0.97, *P*=0.023), *Prevotellaceae* (OR=0.8, 95%CI:0.68-0.99, *P*=0.043), *Bifidobacterium* (OR=0.83, 95%CI:0.72-0.95, *P*=0.009), *Actinomyces* (OR=0.8, 95%CI:0.72-0.9, *P*=0.040), *RikenellaceaeRC9* (OR=0.85, 95%CI:0.75-0.95, *P*=0.005), *Holdemania* (OR=0.87, 95%CI:0.76-0.98, *P*=0.02). 2 classes, 1 order, 1 family, and 3 genera of gut microbiota have been identified as risk factors for the occurrence of nasal polyps, including *Methanobacteria* (OR=1.13, 95%CI:1.00-1.27, *P*=0.046), *Deltaproteobacteria* (OR=1.28, 95%CI:1.05-1.56, *P*=0.014), *Methanobacteriales* (OR=1.14, 95%CI:1.01-1.30, *P*=0.042), *Methanobacteriaceae* (OR=1.13, 95%CI:1.00-1.27, *P*=0.046), *Candidatus Soleaferrea* (OR=1.15, 95%CI:1.01-1.30, *P*=0.036), *Eubacterium fissicatena* (OR=1.19, 95%CI:1.06-1.34, *P*=0.004), *Methanobrevibacter* (OR=1.21, 95%CI:1.01-1.45, *P*=0.035). Interestingly, *Methanobacteria*, *Methanobacteriales*, *Methanobacteriaceae*, and *Methanobrevibacter* all belong to the same class, suggesting that this group of gut microbiota may exert a more significant causal effect on nasal polyps. The mr_steiger algorithm indicated that the Mendelian randomization analysis was consistently directional. Additionally, we observed heterogeneity in the *Actinobacteria* results and horizontal pleiotropy in the Bifidobacterium results, which could potentially affect the reliability of the causal estimates. However, after correction for the false discovery rate (FDR), no taxa remained statistically significant (**Figure 3A**).

MR-BMA analysis was conducted on gut microorganisms exhibiting a significant causal association with nasal polyps, enabling the ranking of their importance. *Actinobacteria* and *Bifidobacterium* were excluded from the analysis due to observed heterogeneity or horizontal pleiotropy. Important gut microbial taxa have potential as biomarkers. The results revealed 4 microorganisms with MIP values greater than 0.1: *Candidatus Soleaferrea* (MACE=0.112, MIP=0.427, *P*=0.0104), *Methanobacteria* (MACE=0.026, MIP=0.165, *P*=0.2329), *Eubacterium fissicatena* (MACE=0.010, MIP=0.111, *P*=0.1720), and *RikenellaceaeRC9* (MACE=-0.010, MIP=0.118, *P*=0.1384). *Candidatus Soleaferrea* exhibited the highest posterior probability of 0.427, while the remaining 3 key microbes had posterior probabilities exceeding 0.03. See **Supplementary File S3** for more details. **Table 1** lists all gut microbiota and immune traits with significant P-values from the MR-BMA analysis.

**Table 1:** MR-BMA analysis of gut microbiota and immune traits on polyps.

Category	Exposure	Polyp	Posterior prob	MACE	MIP	*P* value
Gut microbiota	*Candidatus Soleaferrea*	Nasal polyp	0.427	0.111	0.657	0.0104
Gut microbiota	*unknowngenus.id.959*	Gallbladder polyp	0.193	0.311	0.670	0.0078
Gut microbiota	*Actinobacteria*	Colon polyp	0.445	-0.070	0.599	0.0246
Gut microbiota	*Gastranaerophilales*	Gastric polyp	0.201	0.263	0.935	0.0011
Gut microbiota	*Eggerthella*	Gastric polyp	0.002	0.137	0.538	0.0244
B cell	CD27 on switched memory B cell	Nasal polyp	0.805	-0.035	0.846	0.0016
B cell	CD20 on CD20- CD38- B cell	Gallbladder polyp	0.327	-0.092	0.531	0.0357
B cell	CD27 on T cell	Colon polyp	0.962	0.031	0.974	0.0011
cDC	CCR2 on CD62L^+^ myeloid Dendritic Cell	Nasal polyp	0.931	-0.052	0.944	0.0169
cDC	CD62L- monocyte Absolute Count	Gallbladder polyp	0.592	0.188	0.759	0.0366
Maturation stages of T cell	Terminally Differentiated CD4^+^ T cell %T cell	Nasal polyp	0.336	-0.014	0.356	0.0448
Maturation stages of T cell	CD8 on naive CD8^+^ T cell	Gallbladder polyp	0.662	0.105	0.733	0.0357
Maturation stages of T cell	Terminally Differentiated CD4-CD8- T cell %CD4-CD8- T cell	Gastric polyp	0.04	-0.114	0.998	0.0002
Maturation stages of T cell	Naive CD4-CD8- T cell %T cell	Gastric polyp	0.001	0.056	0.950	0.0004
Myeloid cell	CD14 on Monocytic Myeloid-Derived Suppressor Cells	Gallbladder polyp	0.727	0.156	0.831	0.0278
Myeloid cell	HLA DR on CD33^+^ HLA DR^+^ CD14dim	Colon polyp	0.919	0.025	0.933	0.0048
Myeloid cell	CD33 on CD33^+^ HLA DR^+^ CD14dim	Gastric polyp	0.209	0.005	0.230	0.0256
Myeloid cell	CD33 on CD33^+^ HLA DR^+^	Gastric polyp	0.135	0.007	0.176	0.0245
Myeloid cell	CD33 on Monocytic Myeloid-Derived Suppressor Cells	Gastric polyp	0.13	0.004	0.167	0.0338
TBNK	SSC-A on lymphocyte	Gallbladder polyp	0.456	0.100	0.536	0.0392
Treg	CD45RA on CD39^+^ resting CD4 regulatory T cell	Nasal polyp	0.295	0.012	0.316	0.0461
Treg	CD28- CD8^+^ T cell %T cell	Gallbladder polyp	0.224	-0.190	0.454	0.0259
Treg	CD25^++^ CD45RA^+^ CD4 not regulatory T cell %T cell	Colon polyp	0.436	-0.004	0.439	0.0350
Treg	CD28- CD8dim T cell %T cell	Gastric polyp	0.698	0.054	0.777	0.0006

MIP, marginal inclusion probability; MACE, model-averaged causal effect.

#### 3.2.2 Univariate MR analysis and MR-BMA analysis of immune traits on nasal polyp

**Figure 4:**
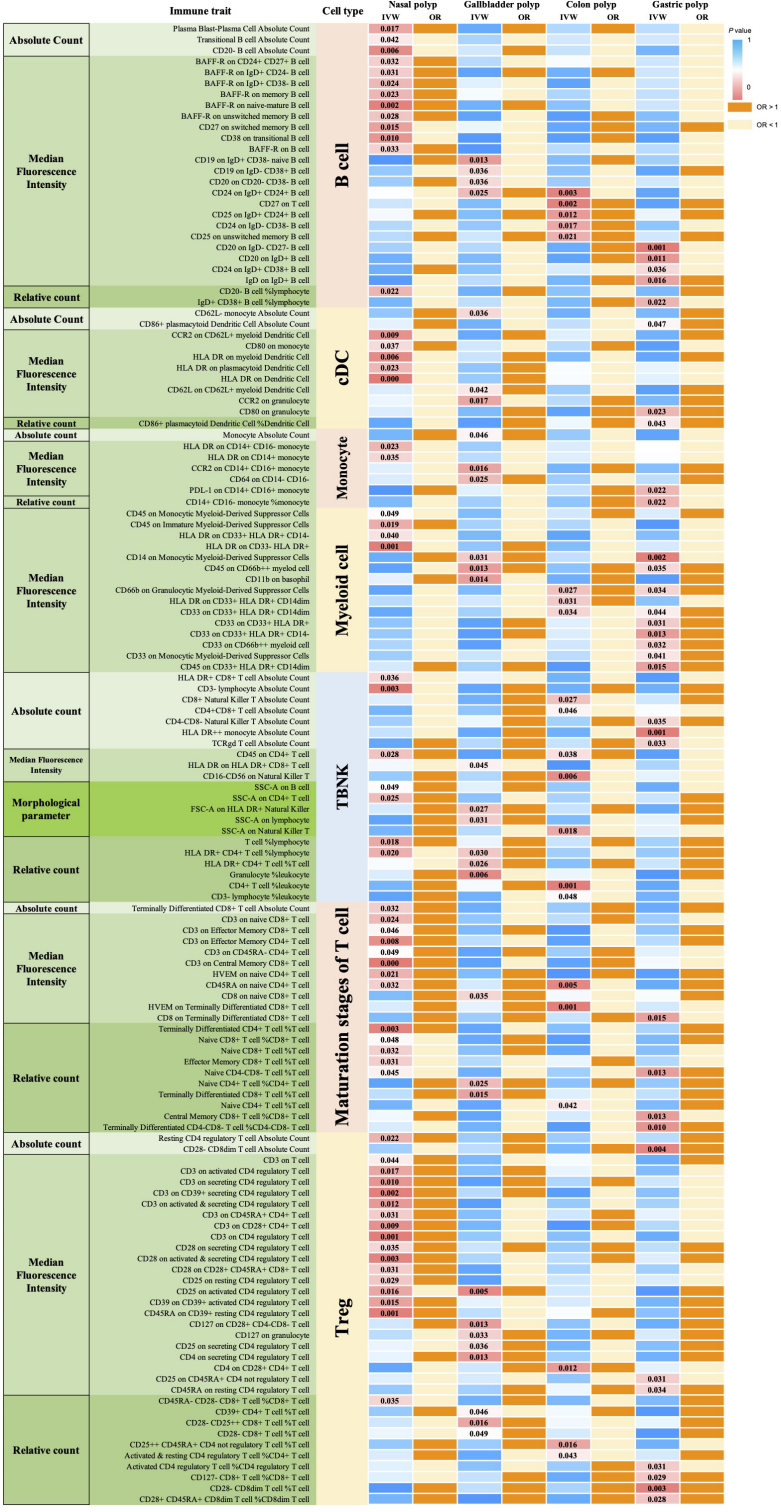
MR analysis of 731 immune traits for causal effects on 4 polyp types. 135 immune traits had significant causal effects on at least 1 polyp type. P-values for immune traits with significant causal effects associated with B cells (26) Dendritic cells (11), Monocyte (7), Myeloid cell (15), TBNK (21), Maturation stages of T cells (21), and Treg (34) are indicated. OR greater than 1 indicates that the immune trait is a risk factor for nasal polyps, while OR less than 1 indicates a protective effect.

According to IVW method, we identified a total of 135 immune traits that were significantly causally associated with at least one type of polyp, of which 61 immune traits were potentially associated with nasal polyps (**Figure 4**). Based on the immune cell types, they were specifically classified into 13 traits of B cells, 5 traits of cDC, 13 traits of Maturation stages of T cells, 2 traits of Monocyte, 4 traits of Myeloid cells, 7 traits of TBNK and 17 traits of Treg. Mr_steiger algorithm indicated that the MR analysis did not exhibit significant bidirectionality. Among these 61 immune traits, 35 did not exhibit heterogeneity or pleiotropy. After multiple test correction and sensitivity analysis, only two immune traits remained statistically significant (*P*_FDR_ < 0.05). The significant immune traits were: CD3 on Central Memory CD8^+^ T cells (OR=1.13, 95%CI:1.06-1.21, *P*_FDR_=0.012), which belongs to the T cell maturation stage category, and CD3 on CD4 regulatory T cells (OR=1.13, 95%CI:1.06-1.20, *P*_FDR_=0.047), which belongs to Treg **Figure 3B**.

We conducted MR-BMA analyses on immune traits exhibiting significant causal associations with nasal polyps, providing insights into the true importance of these immune traits for nasal polyp development. Among the 61 immune traits with significant causality, 26 exhibited heterogeneity or horizontal pleiotropy and were therefore excluded from the MR-BMA analysis. Consistent with the categorization in the univariate MR analysis, we classified the remaining immune traits into 7 categories for the MR-BMA analysis and ranked their significance based on MIP values. See **Supplementary File S3** for details. 4 immune traits were identified as significant, with MIP and posterior probability greater than 0.2: CD27 on switched memory B cells (MACE=-0.035, MIP=0.846, *P*=0.0016), CCR2 on CD62L^+^ myeloid dendritic cells (MACE=-0.052, MIP=0.944, *P*=0.0169), Terminally Differentiated CD4^+^ T cell %T cell (MACE=-0.014, MIP=0.356, *P*=0.0448), with posterior probability models of 0.805, 0.931, and 0.336, respectively. Additionally, CD45RA on CD39^+^ resting CD4 regulatory T cells (MACE=0.012, MIP=0.316, *P*=0.0461) exhibited a posterior probability of 0.295 **Table 1**.

#### 3.2.3 Mediated Mendelian randomization analysis

Through univariate MR analysis, we identified a total of 11 gut microbial taxa and 35 immune traits exhibiting significant causal effects on nasal polyps, without evidence of heterogeneity or horizontal pleiotropy. Subsequently, we conducted a two-sample MR analysis to investigate the relationships between these 11 gut microbes and 35 immune traits. See **Supplementary File S4** for details. We identified a total of 11 causal pathways through which gut microbiota modulate immune function, thereby influencing the development of nasal polyps. The mediated proportion of these immune traits was also obtained. Among these, the mediating direction of 6 causal pathways (Beta1×Beta2) was consistent with the direction of the gut microbe-nasal polyp association (Beta3). *Methanobrevibacter* inhibited CCR2 on CD62L^+^ myeloid dendritic cells, a protective factor against nasal polyps, or mediated elevated levels of CD3 on CD4 regulatory T cells, which ultimately led to an increased risk of polyps. *Prevotellaceae* increased the risk of nasal polyps by decreasing CD39 on CD39^+^ activated CD4 regulatory T cells and CD45RA on naive CD4^+^ T cells, which are protective factors against nasal polyps. Additionally, *Eubacterium fissicatena* and *Deltaproteobacteria* affected Effector Memory CD8^+^ T cell %T cell and Terminally Differentiated CD8^+^ T cell Absolute Count, respectively, thereby increasing the risk of polyp. Notably, *Eubacterium fissicatena* was identified as a key microbial taxa in the MR-BMA analysis, suggesting that the mechanism by which *Eubacterium fissicatena* mediates Effector Memory CD8^+^ T cell %T cell levels may play an important role in the development of nasal polyps **Table 2**.

**Table 2:** Analysis of immune traits as mediators of gut microbiota to polyps.

Exposure	Mediator	Outcome	Beta1	Beta2	Beta3	Mediator effect	Mediated Proportion
*Methanobrevibacter*	CCR2 on CD62L^+^ myeloid Dendritic Cell	Nasal polyp	-0.211	-0.076	0.194	0.016	8.33%
*Holdemania*	CCR2 on CD62L^+^ myeloid Dendritic Cell	Nasal polyp	-0.200	-0.076	-0.144	0.015	-10.57%
*Candidatus Soleaferrea*	CD25 on activated CD4 regulatory T cell	Nasal polyp	0.248	-0.112	0.136	-0.028	-20.31%
*Methanobrevibacter*	CD3 on CD4 regulatory T cell	Nasal polyp	0.260	0.118	0.194	0.031	15.88%
*RikenellaceaeRC9*	CD39 on CD39^+^ activated CD4 regulatory T cell	Nasal polyp	0.163	0.041	-0.168	0.007	-3.96%
*Prevotellaceae*	CD39 on CD39^+^ activated CD4 regulatory T cell	Nasal polyp	-0.248	0.041	-0.198	-0.010	5.13%
*Prevotellaceae*	CD45RA on naive CD4^+^ T cell	Nasal polyp	-0.288	0.035	-0.198	-0.010	5.05%
*Eubacterium fissicatena*	Effector Memory CD8^+^ T cell %T cell	Nasal polyp	-0.173	-0.047	0.173	0.008	4.74%
*Deltaproteobacteria*	Effector Memory CD8^+^ T cell %T cell	Nasal polyp	0.274	-0.047	0.246	-0.013	-5.28%
*Deltaproteobacteria*	Terminally Differentiated CD8^+^ T cell Absolute Count	Nasal polyp	0.310	0.065	0.246	0.020	8.17%
*Prevotellaceae*	Transitional B cell Absolute Count	Nasal polyp	-0.231	-0.059	-0.198	0.014	-6.94%
*Peptococcaceae*	CD14 on Monocytic Myeloid-Derived Suppressor Cells	Gallbladder polyp	-0.325	0.114	0.533	-0.037	-6.94%
*unknowngenus.id.959*	SSC-A on lymphocyte	Gallbladder polyp	-0.189	0.265	-0.713	-0.050	7.02%
*RuminococcaceaeUCG002*	CD24 on IgD- CD38- B cell	Colon polyp	0.222	0.024	0.086	0.005	6.20%
*RuminococcaceaeUCG002*	CD24 on IgD^+^ CD24^+^ B cell	Colon polyp	0.304	-0.027	0.086	-0.008	-9.42%
*Eubacteriumbrachy*	CD4 on CD28^+^ CD4^+^ T cell	Colon polyp	-0.207	0.039	-0.067	-0.008	12.11%
*Actinobacteria*	CD28^+^ CD45RA^+^ CD8dim T cell %CD8dim T cell	Gastric polyp	0.279	0.031	0.263	0.009	3.24%
*Actinobacteria*	CD66b on Granulocytic Myeloid-Derived Suppressor Cells	Gastric polyp	-0.297	0.046	0.263	-0.014	-5.20%
*Actinobacteria*	PDL-1 on CD14^+^ CD16^+^ monocyte	Gastric polyp	-0.265	-0.046	0.263	0.012	4.59%
*Eggerthella*	CD33 on CD33^+^ HLA DR^+^	Gastric polyp	-0.336	0.028	0.181	-0.010	-5.28%
*Eggerthella*	CD86^+^ plasmacytoid Dendritic Cell %Dendritic Cell	Gastric polyp	-0.169	0.063	0.181	-0.011	-5.83%
*Eubacterium oxidoreducens*	CD20 on IgD- CD27- B cell	Gastric polyp	0.285	-0.114	-0.262	-0.032	12.40%
*Eubacterium oxidoreducens*	CD28- CD8dim T cell %T cell	Gastric polyp	0.282	0.070	-0.262	0.020	-7.55%
*Eubacterium oxidoreducens*	CD45 on CD66b^++^ myeloid cell	Gastric polyp	-0.270	0.065	-0.262	-0.018	6.70%
*Gastranaerophilales*	CD45 on CD66b^++^ myeloid cell	Gastric polyp	0.214	0.065	-0.156	0.014	-8.93%
*Gastranaerophilales*	CD8 on Terminally Differentiated CD8^+^ T cell	Gastric polyp	0.217	-0.068	-0.156	-0.015	9.46%
*unknownfamily.id.1000001214*	CD45 on CD66b^++^ myeloid cell	Gastric polyp	0.214	0.065	-0.156	0.014	-8.93%
*unknownfamily.id.1000001214*	CD8 on Terminally Differentiated CD8^+^ T cell	Gastric polyp	0.217	-0.068	-0.156	-0.015	9.46%
*unknowngenus.id.1000001215*	CD45 on CD66b^++^ myeloid cell	Gastric polyp	0.214	0.065	-0.156	0.014	-8.93%
*unknowngenus.id.1000001215*	CD8 on Terminally Differentiated CD8^+^ T cell	Gastric polyp	0.217	-0.068	-0.156	-0.015	9.46%

### 3.3 Causal relationships between the gut microbiota, immune traits and gallbladder polyp

#### 3.3.1 Univariate MR analysis and MR-BMA analysis of gut microbiota on gallbladder polyp

**Figure 5:**
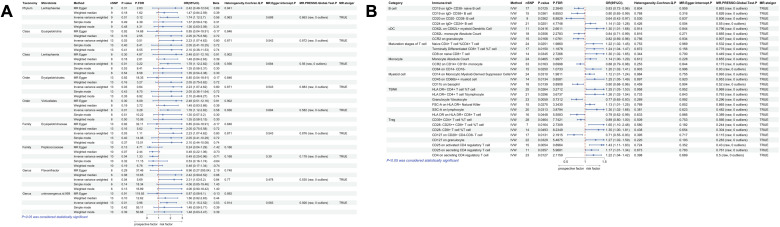
Univariate MR results of gut microbiota and immune traits on gallbladder polyps. **(A)** MR forest plot of the causal effect of gut microbiota on gallbladder polyps. **(B)** MR forest plot of the causal effect of immune traits on gallbladder polyps. OR>1 indicates an increased risk, whereas OR<1 indicates a decreased risk. The last 4 columns are analyses of horizontal pleiotropy, heterogeneity, and the directionality of MR, with *P* > 0.05 indicating no pleiotropy or heterogeneity and TRUE indicating no reverse causality.

According to the IVW method of univariate MR we identified a total of 9 gut microbial taxa that may be associated with gallbladder polyps (**Figure 2**). Among them, 1 family was a protective factor for the development of gallbladder polyps, *Peptococcaceae* (OR=0.49, 95%CI:0.25-0.96, *P*=0.038). 1 phylum, 2 orders, 2 orders, 1 family, and 2 genera were risk factors for the development of nasal polyps. *Lentisphaerae* (OR=1.74, 95%CI:1.12-2.70, *P*=0.013), *Lentisphaeria* (OR=1.78, 95%CI:1.12-2.82, *P*=0.014), *Erysipelotrichia* (OR=2.23, 95%CI:1.07-4.62, *P*=0.032), *Erysipelotrichales* (OR=2.23, 95%CI:1.07-4.62, P=0.032), *Victivallales* (OR=1.78, 95%CI:1.12-2.82, *P*=0.014), *Erysipelotrichaceae* (OR=2.23, 95%CI:1.07-4.62, *P*=0.032), *Flavonifractor* (OR=2.31, 95%CI:1.03-5.20, *P*=0.044), *unknowngenus.id.959* (OR=1.70, 95%CI:1.15-2.52, *P*=0.007). No heterogeneity or pleiotropy was observed in any of the taxa. *Lentisphaeria* and *Lentisphaerae* belonged to one taxonomic group, while *Erysipelotrichia*, *Erysipelotrichales*, and *Erysipelotrichaceae* belonged to another group. These two groups might have a more significant causal effect. Mr_steiger algorithm showed consistent directionality in the MR analysis. After correcting for FDR, no microbiota reached statistical significance (**Figure 5A**).

To understand the true importance of gut microorganisms causally associated with gallbladder polyps, we performed MR-BMA analysis. 8 microorganisms had MIP values greater than 0.1: *unknowngenus.id.959* (MACE=0.311, MIP=0.670, *P*=0.0078), *Lentisphaerae* (MACE=0.057, MIP=0.176, P=0.1440), *Lentisphaeria* (MACE=0.053, MIP=0.168, *P*=0.1638), *Victivallales* (MACE=0.053, MIP=0.168, *P*=0.1638), *Erysipelotrichales* (MACE=0.037, MIP=0.120, *P*=0.8759), *Erysipelotrichia* (MACE=0.037, MIP=0.120, *P*=0.8759), *Erysipelotrichaceae* (MACE=0.037, MIP=0.120, *P*=0.8759), and *Peptococcaceae* (MACE=-0.142, MIP=0.290, *P*=0.0847) (**Supplementary File S3**). *Unknowngenus.id.959* had the highest posterior probability of 0.193. See **Table 1** for detail.

#### 3.3.2 Univariate MR analysis and MR-BMA analysis of immune traits on gallbladder polyp

Using IVW method of univariate MR, we identified a total of 30 immune traits potentially associated with gallbladder polyps (**Figure 4**). These included 4 traits of B cell, 3 traits of cDC, 3 traits of Maturation stages of T cell, 3 traits of Monocyte, 3 traits of Myeloid cell, 6 traits of TBNK and 8 traits of Treg. Mr_steiger algorithm indicated that the MR analysis did not show significant bidirectionality. None of the 30 immune traits exhibited heterogeneity or pleiotropy. After correcting the p-values for multiple testing using FDR, no immune traits remained statistically significant (*P* < 0.05 after FDR adjustment) (**Figure 5B**).

We also performed MR-BMA analysis on the 30 immune traits to gain further insight into their true significance for gallbladder polyps, as this method accounts for potential causal associations. Consistent with the univariate MR analysis, we categorized the immune traits into 7 classes for the MR-BMA analysis and ranked their importance based on MIP values (**Supplementary File S3**). The posterior probability models for CD20 on CD20- CD38- B cell (MACE=-0.092, MIP=0.531, *P*=0.0357), CD28- CD8^+^ T cell %T cell (MACE=-0.190, MIP=0.454, *P*=0.0259) reached 0.327, and 0.224, respectively. CD62L- monocyte Absolute Count (MACE=0.188, MIP=0.759, *P*=0.0366), CD8 on naive CD8^+^ T cell (MACE=0.105, MIP=0.733, *P*=0.0357), CD14 on Monocytic Myeloid-Derived Suppressor Cells (MACE=0.156, MIP=0.831, *P*=0.0278), and SSC-A on lymphocyte (MACE=0.100, MIP=0.536, *P*=0.0392) with posterior probabilities of 0.592, 0.662 0.727 and 0.456. **Table 1**. The posterior probability of each of these immune traits exceeds 0.2 and is a crucial player in the development of gallbladder polyps.

#### 3.3.3 Mediated Mendelian randomization analysis

Using univariate MR, we identified 9 gut microbial taxa and 30 immune traits that had a significant causal effect on gallbladder polyps, without evidence of heterogeneity or horizontal pleiotropy. We then performed a two-sample MR analysis to investigate the relationship between these 9 gut microbial taxa and the 30 immune traits (**Supplementary File S4**). Two causal pathways were identified, through which gut microbiota modulate immune function and consequently influence the development of gallbladder polyps. The mediated proportion of these immune traits was also obtained. One of these causal pathways had a mediating direction (Beta1×Beta2) that was consistent with the direction of the effect of gut microbiota on gallbladder polyps (Beta3). The gut microbe *unknowngenus.id.959* acts by inhibiting SSC-A (a marker) on lymphocytes, which is a risk factor for colon polyps. Notably, *unknowngenus.id.959* was the most important gut microbe identified in the MR-BMA analysis. Therefore, the mechanism by which *unknowngenus.id.959* mediates the level of SSC-A on lymphocytes may be an important protective mechanism against gallbladder polyps **Table 2**.

### 3.4 Causal relationships between the gut microbiota, immune traits and colon polyp

#### 3.4.1 Univariate MR analysis and MR-BMA analysis of gut microbiota on colon polyp

**Figure 6:**
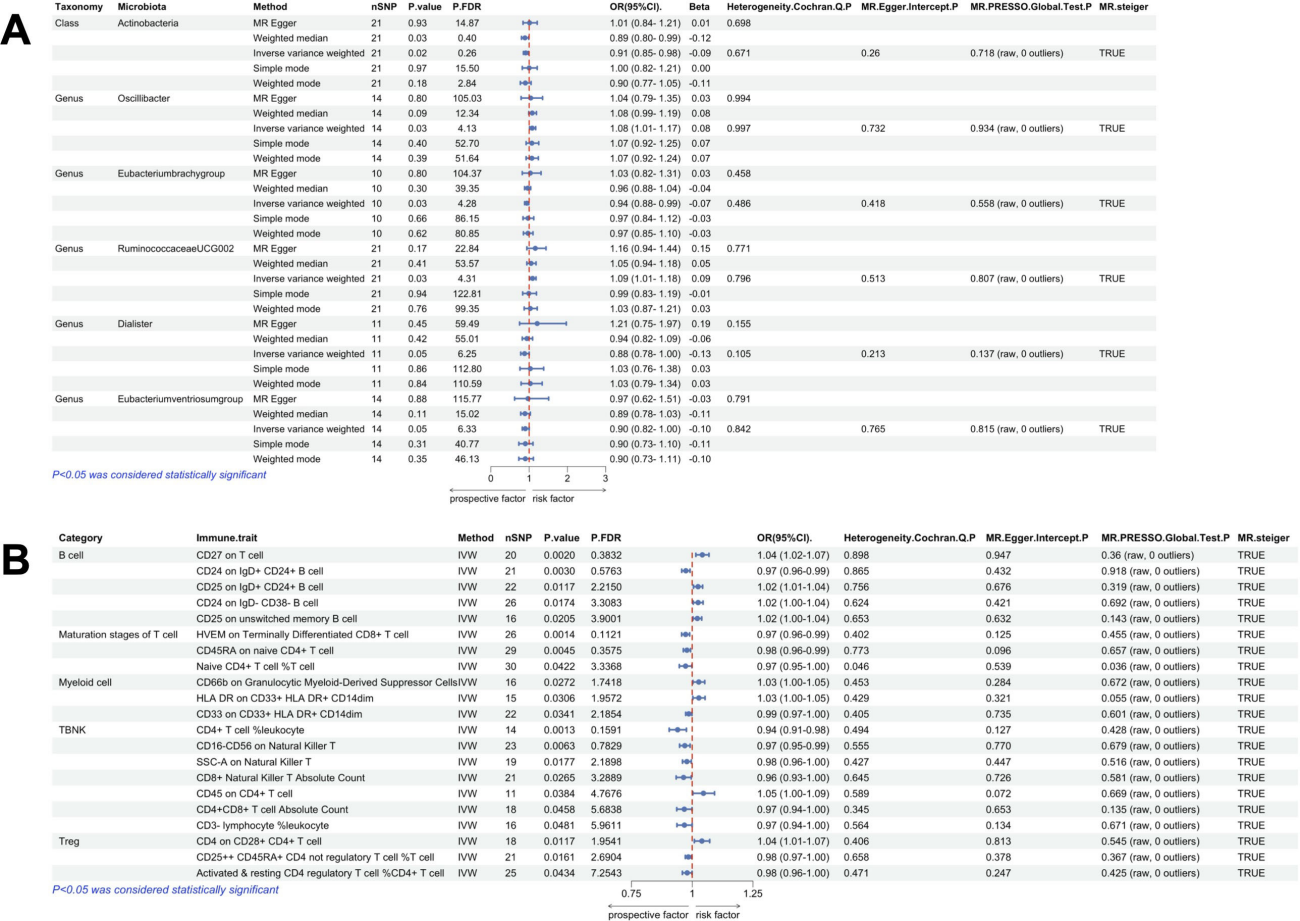
Univariate MR results of gut microbiota and immune traits on colon polyps. **(A)** MR forest plot of the causal effect of gut microbiota on colon polyps. **(B)** MR forest plot of the causal effect of immune traits on colon polyps. OR>1 indicates an increased risk, whereas OR<1 indicates a decreased risk. The last 4 columns are analyses of horizontal pleiotropy, heterogeneity, and the directionality of MR, with *P* > 0.05 indicating no pleiotropy or heterogeneity and TRUE indicating no reverse causality.

Using IVW method of Mendelian randomization, we identified 6 gut microbial taxa potentially associated with colon polyps (**Figure 2**). *Oscillibacter* (OR=1.08, 95%CI:1.01-1.17, *P*=0.032) and *RuminococcaceaeUCG002* (OR=1.09, 95%CI:1.01-1.18, *P*=0.033) were identified as having a potentially positive causative effect, increasing the risk of colon polyps. In contrast, *Dialister* (OR=0.88, 95%CI:0.78-1.00, *P*=0.048), *Eubacteriumventriosum* (OR=0.90, 95%CI:0.82-1.00, *P*=0.048), *Actinobacteria* (OR=0.91, 95%CI:0.85-0.98, *P*=0.016), and *Eubacterium brachy* (OR=0.94, 95%CI:0.88-0.99, *P*=0.033) were associated with a reduced risk of colon polyps. Mr_steiger algorithm did not reveal an inverse causal relationship between these 6 taxa and colon polyps. However, none of the results remained statistically significant after correcting for multiple testing using FDR method **Figure 6A**.

MR-BMA analysis of gut microorganisms with significant causative associations for colon polyps provides further insight into their significance. MIP values of 4 microorganisms greater than 0.1: *Actinobacteria* (MACE=-0.070, MIP=0.599, *P*=0.0246), *Oscillibacter* (MACE=0.012, MIP=0.145, *P*=0.3453), *Eubacteriumventriosum* (MACE=-0.029, MIP=0.255, *P*=0.2027), and *Dialister* (MACE=-0.010, MIP=0.105, *P*=0.8050) (**Supplementary File S3**). *Actinobacteria* had the highest posterior probability of 0.445. See **Table 1** for detailed results.

#### 3.4.2 Univariate MR analysis and MR-BMA analysis of immune traits on colon polyp

Using IVW method of univariate MR, we identified a total of 21 immune traits potentially associated with colon polyps (**Figure 4**). These included 5 traits of B cell, 3 traits of Maturation stages of T cell, 3 traits of Myeloid cell, 7 traits of TBNK and 3 traits of Treg. Mr_steiger algorithm indicated that MR analysis did not show significant bidirectionality. However, the Cochran’s Q test for heterogeneity and the MR-PRESSO test revealed some heterogeneity in the Naive CD4^+^ T cell %T cell, which affected the causality results. After correcting the p-values for FDR method, no immune trait remained statistically significant (*P* < 0.05 after FDR adjustment) (**Figure 6B**).

The 20 immune traits that had a significant causal effect on colon polyps were subjected to MR-BMA analysis to gain insight into their true importance for colon polyps. Consistent with the univariate MR analysis, we categorized these immune traits into 7 classes for the MR-BMA analysis and ranked their importance based on MIP values (**Supplementary File S3**). The immune traits CD27 on T cell (MACE=0.031, MIP=0.974, *P*=0.0011) and HLA DR on CD33^+^ HLA DR^+^ CD14dim (MACE=0.025, MIP=0.933, *P*=0.005) had posterior probabilities of 0.962 and 0.919, respectively. The immune trait cd25^++^ CD45RA^+^ CD4 not regulatory T cell %T cell (MACE=-0.004, MIP=0.439, *P*=0.0350) had a posterior probability of 0.436. These immune traits play an important role in the development of colon polyps.

#### 3.4.3 Mediated Mendelian randomization analysis

Using univariate MR, we identified 6 gut microbial taxa and 20 immune traits that had a significant causal effect on colon polyps, without evidence of heterogeneity or horizontal pleiotropy. Two-sample MR analysis was performed to investigate the relationship between these 6 gut microbial taxa and the 20 immune traits (**Supplementary File S4**). We identified two causal pathways through which gut microbes modulate immune function and consequently influence the development of colon polyps, and obtained the mediated proportion for these immune traits. Among these, the mediating direction (Beta1×Beta2) of three causal pathways was consistent with the direction of the effect of gut microbes on colon polyps (Beta3). Increased abundance of *RuminococcaceaeUCG002* can cause increased levels of CD24 on IgD- CD38- B cells, which in turn leads to an increased risk of colon polyps. Conversely, Eubacterium brachy may reduce the risk of colon polyps by decreasing the levels of CD4 on CD28^+^ CD4^+^ T cells (**Table 2**).

### 3.5 Causal relationships between the gut microbiome, immune traits and gastric polyp

#### 3.5.1 Univariate MR analysis and MR-BMA analysis of gut microbiota on gastric polyp

**Figure 7:**
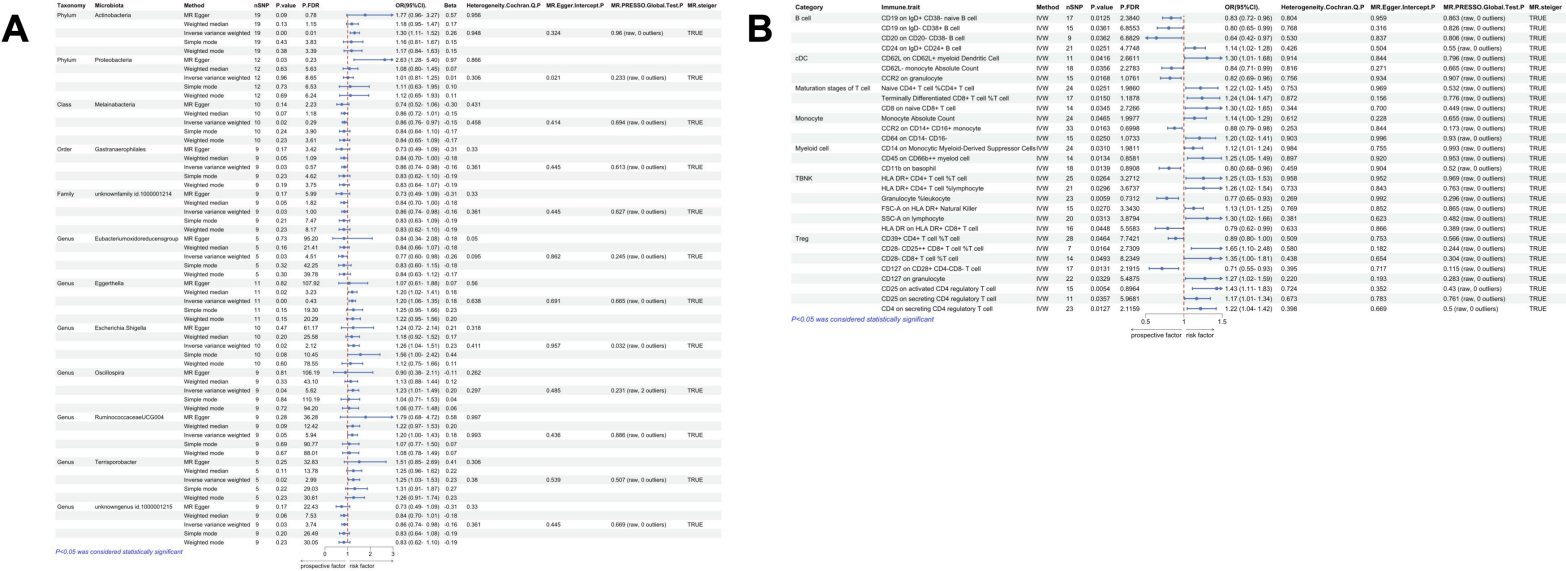
Univariate MR results of gut microbiota and immune traits on gastric polyps. **(A)** MR forest plot of the causal effect of gut microbiota on gastric polyps. **(B)** MR forest plot of the causal effect of immune traits on gastric polyps. OR>1 indicates an increased risk, whereas OR<1 indicates a decreased risk. The last 4 columns are analyses of horizontal pleiotropy, heterogeneity, and the directionality of MR, with *P* > 0.05 indicating no pleiotropy or heterogeneity and TRUE indicating no reverse causality.

We identified a total of 12 gut microbial taxa potentially associated with gastric polyps by IVW method **Figure 2**. There was evidence of horizontal pleiotropy and heterogeneity for the taxa *Oscillospira* and *Proteobacteria*. *RuminococcaceaeUCG004* (OR=1.12, 95%CI:1.00-1.43, *P*=0.045), *Eggerthella* (OR=1.20, 95%CI:1.06-1.35, P=0.003), *Terrisporobacter* (OR=1.25, 95%CI:1.03-1.53, *P*=0.023), *Escherichia.Shigella* (OR=1.26, 95%CI:1.04-1.51, *P*=0.016), and *Actinobacteria* (OR=1.30, 95%CI:1.11-1.52, *P*=0.001) were identified as having a positive causal effect on gastric polyps, potentially increasing the risk. In contrast, E*ubacterium oxidoreducens* (OR=0.77, 95%CI:0.60-0.98, *P*=0.034), *Gastranaerophilales* (OR=0.86, 95%CI:0.74-0.98, *P*=0.029), *unknownfamily.id.1000001214* (OR=0.86, 95%CI:0.74-0.98, *P*=0.029), *unknowngenus.id.1000001215* (OR=0.86, 95%CI:0.74-0.98, *P*=0.029), and *Melainabacteria* (OR=0.86, 95%CI:0.76-0.97, *P*=0.018) were associated with a reduced risk of gastric polyps. Mr_steiger algorithm did not reveal any evidence of reverse causality between 10 microbial taxa and gastric polyps. *Actinobacteria* remained statistically significant after correcting for multiple testing using FDR method (FDR-adjusted *P* < 0.05) **Figure 7A**.

We performed MR-BMA analysis on the gut microorganisms that were significantly causally associated with gastric polyps to gain further insight into their significance. 7 microorganisms had MIP values greater than 0.1: *Gastranaerophilales* (MACE=0.263, MIP=0.935, *P*=0.0011), *Eggerthella* (MACE=0.137, MIP=0.538, *P*=0.0244), *Actinobacteria* (MACE=-0.025, MIP=0.180, *P*=0.0729), *Melainabacteria* (MACE=-0.023, MIP=0.173, *P*=0.0822), *Terrisporobacter* (MACE=-0.023, MIP=0.173, *P*=0.0822), *unknowngenus id.1000001215* (MACE=-0.023, MIP=0.173, *P*=0.0822), and *unknownfamily id.1000001214* (MACE=0.022, MIP=0.172, *P*=0.1138). **Supplementary File S3**. 7 *Gastranaerophilales* had the highest posterior probability of 0.201. See **Table 1** for detailed results.

#### 3.5.2 Univariate MR analysis and MR-BMA analysis of immune traits on gastric polyp

According to the IVW method of univariate MR we identified a total of 33 immune traits that may has causal relationship with gastric polyps **Figure 4**. These included 5 traits of B cell, 3 traits of cDC, 4 traits of Maturation stages of T cell, 2 traits of Monocyte, 9 traits of Myeloid cell, 3 traits of TBNK and 7 traits of Treg. Mr_steiger algorithm indicated that the MR analysis did not show significant bidirectionality. Sensitivity analysis did not reveal heterogeneity and horizontal pleiotropy in these 33 immune traits. After correcting the p-values for multiple testing using FDR method, no immune trait remained statistically significant (*P* < 0.05 after FDR adjustment) **Figure 7B**.

Consistent with the univariate MR analysis, we categorized these immune traits into 7 classes for the MR-BMA analysis and ranked their importance based on MIP values **Supplementary File S3**. A total of 6 immune traits had MIP > 0.1 and p-value < 0.05, including Terminally Differentiated CD4-CD8- T cell %CD4-CD8- T cell (MACE=-0.114, MIP=0.998, *P*=0.0002), Naive CD4-CD8- T cell %T cell (MACE=0.056, MIP=0.950, *P*=0.0004), CD28- CD8dim T cell %T cell (MACE=0.054, MIP=0.777, *P*=0.0006), CD33 on CD33^+^ HLA DR^+^ (MACE=0.007, MIP=0.176, *P*=0.0245), CD33 on CD33^+^ HLA DR^+^ CD14dim (MACE=0.005, MIP=0.230, *P*=0.0256), and CD33 on Monocytic Myeloid-Derived Suppressor Cells (MACE=0.004, MIP=0.167, *P*=0.0338).

#### 3.5.3 Mediated Mendelian randomization analysis

By univariate MR, we found a total of 10 gut microbial taxa as well as 33 immune traits to have significant causal effects on gallbladder polyps without heterogeneity or horizontal pleiotropy. Two-sample MR analysis was performed to investigate the relationship between these 10 gut microbial taxa and the 33 immune traits **Supplementary File S4**. We identified a total of 14 causal pathways through which gut microbes modulate immune function and consequently influence the development of gastric polyps, and obtained the mediated proportion for these immune traits. Among these, the mediating direction (Beta1×Beta2) of 7 causal pathways was consistent with the direction of the effect of gut microbes on gastric polyps (Beta3). *Actinobacteria* induced polyp development by inhibiting PDL-1 on CD14^+^ CD16^+^ monocytes or increasing the percentage of CD28^+^ CD45RA^+^ CD8dim T cells of CD8dim T cells. *Eubacterium oxidoreducens* induced polyp development by mediating the levels of CD45 on CD66b^++^ myeloid cells and CD20 on IgD- CD27- B cells, which exert a suppressive effect on polyp development. *Gastranaerophilales* may reduce the risk of gastric polyps by increasing the level of CD8 on terminally differentiated CD8^+^ T cells. Additionally, *unknownfamily.id.1000001214* and *unknowngenus.id.1000001215* may exert their protective effects through CD8 on terminally differentiated CD8^+^ T cells **Table 2**. Based on MR-BMA results, *Gastranaerophilales*, *Actinobacteria*, *unknowngenus id.1000001215*, and *unknownfamily id.1000001214* are key gut microbes that play an important protective role against gastric polyps.

## 4 Discussion

**Figure 8:**
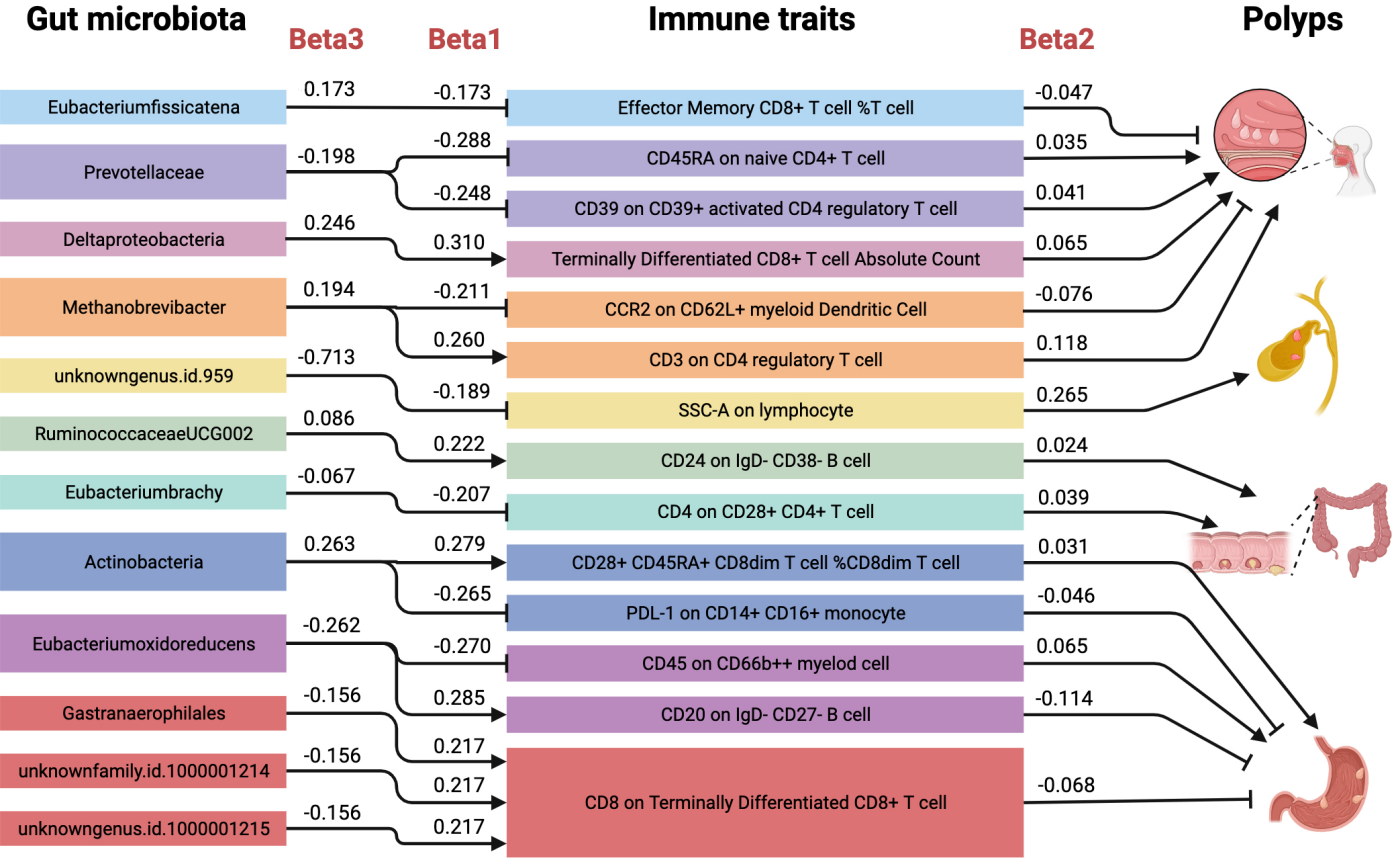
Mediating role of immune traits in the causal effect of gut microbiota on polyps. Causal effects of gut microbiota on polyps (Beta3), gut microbiota on immune traits (Beta1), and immune traits on polyps (Beta2). There were a total of 16 causal pathways where the mediating direction (Beta1*Beta2) was consistent with the direction of gut microbiota-polyps (Beta3).

In this large-scale, comprehensive MR analysis, we identified 11 microbial taxa with 35 immune traits causally associated with the risk of nasal polyps. 9 microbial taxa with 30 immune traits causally associated with the risk of gallbladder polyps.6 microbial taxa with 20 immune traits causally associated with the risk of colon polyps.10 microbial taxa with 33 immune traits causally associated with the risk of nasal polyps. 10 microbial taxa and 33 immune profiles were causally associated with the risk of nasal polyps. In addition, the MR-BMA results prioritized the importance of these factors. A total of 16 causal pathways were identified, in which the mediating direction (Beta1×Beta2) was consistent with the direction of the effect of gut microbes on polyps (Beta3), as shown in **Figure 8**. Among them, 6 causal pathways were found to be associated with the mechanism of nasal polyp development, including *Eubacterium fissicatena*- Effector Memory CD8^+^ T cell %T cell- Nasal polyp, mediated effect 4.74%; *Prevotellaceae*- CD45RA on naive CD4 ^+^ T cell- Nasal polyp, mediator effect 5.05%; *Prevotellaceae*- CD39 on CD39^+^ activated CD4 regulatory T cell- Nasal polyp, mediator effect 5.13%; *Deltaproteobacteria*- Terminally Differentiated CD8^+^ T cell Absolute Count- Nasal polyp, mediating effect 8.17%; *Methanobrevibacter*- CCR2 on CD62L^+^ myeloid Dendritic Cell- Nasal polyp with a mediator effect of 8.33%; *Methanobrevibacter*- CD3 on CD4 regulatory T cell- Nasal polyp with a mediator effect of 15.88%. 1 causal pathway was found to be associated with the mechanism of gallbladder polypogenesis was *unknowngenus.id.959*- SSC-A on lymphocyte- Gallbladder polyp with a mediating effect of 7.02%. 2 causal pathways were found to be associated with the mechanism of colon polyp development including *RuminococcaceaeUCG002*- CD24 on IgD- CD38- B cell- Colon polyp with a mediating effect of 6.20%; *Eubacterium brachy*- CD4 on CD28^+^ CD4^+^ T cell- Colon polyp, with a mediating effect of 12.11%. 7 causal pathways were found to be associated with the mechanism of gastric polyp development, including *Actinobacteria*- CD28^+^ CD45RA^+^ CD8dim T cell %CD8dim T cell- Gastric polyp with a mediating effect of 3.24%; *Actinobacteria*- PDL-1 on CD14^+^ CD16^+^ monocyte- Gastric polyp with a mediating effect of 4.59%; *Eubacterium oxidoreducens*- CD45 on CD66b^++^ myeloid cell- Gastric polyp, mediating effect 6.70%; *Eubacterium oxidoreducens*- CD20 on IgD- CD27- B cell- Gastric polyp, mediating effect 12.40%; *Gastranaerophilales*, *unknownfamily.id.1000001214*, and *unknowngenus.id.1000001215* all of which inhibited gastric polyp development via CD8 on Terminally Differentiated CD8^+^ T cell, mediating effect 9.46%.

With the advancement of human gut microbial sequencing technology (29), the gut microbial community, a complex and dynamic ecosystem, has emerged as a forefront and a key area of scientific inquiry, underscoring its pivotal role in human health and disease progression. Initially, research concentrated on exploring the potential relationship between gut microbes and digestive diseases, seeking to elucidate the microecosystem’s regulatory mechanisms in digestive physiology and pathological conditions (30–32). As research evolved, the gut microbiota’s broader immunomodulatory role became evident, particularly during immune-mediated and disease-induced immune injury phases, characterized by marked alterations in gut microbiota composition and function (33, 34). Paulos et al. (35) demonstrated that gut microbes regulate CD8^+^ T-cell function via the Toll-like receptor 4 signaling pathway, reinforcing this regulatory mechanism. This tumor-specific immune responses offered fresh insights into tumor immunotherapy. Subsequent studies (36, 37) have extended these findings, underscoring the gut microbiota’s pivotal role in modulating adaptive immune responses, particularly by modulating the function and activity of Th1 and Th17 cell subsets, as well as myeloid-derived cells, which collectively shape the immune response landscape within the tumor microenvironment.

In 1958, American surgeon Eiseman et al. (38) pioneered the use of healthy human fecal enemas to treat four patients with severe postoperative pseudomembranous enterocolitis, introducing gut microbiota regulation as a potential therapeutic approach. The gut microbiota is highly plastic, with its composition influenced by factors such as diet, prebiotics, probiotics, and antibiotics. Recent studies have revealed a strong link between gut microbiota composition and the efficacy of immune checkpoint inhibitors (ICIs). Oral administration of Bifidobacterium bifidum promotes dendritic cell maturation and CD8^+^ T-cell accumulation in tumors, enhancing the efficacy of PD-L1 blockade therapy (39). This observation was further corroborated, wherein transplantation of fecal microbiota from ICI-responsive patients to germ-free mice reduced tumor burden and improved therapeutic outcomes, while fecal microbiota transplantation (FMT) from non-responders showed no such effects (40, 41). Furthermore, microbial metabolism-derived circulating molecules, particularly secondary bile acids and other metabolites, have emerged as key regulators of various immune processes in humans (42). The intricate interplay between the gut microbiota and bile acids represents a pivotal axis in the modulation of immune responses and the pathogenesis of gastrointestinal diseases. The gut microbiota, through its metabolic activities, transforms bile acids, endowing them with the capacity to act as signaling molecules that engage with specific receptors, such as the farnesoid X receptor (FXR) and the G protein-coupled bile acid receptor 1 (TGR5) (43, 44). These receptors, in turn, are implicated in the regulation of inflammation, with activated FXR known to attenuate the nuclear factor kappa-B (NF-κB) signaling pathway, a key mediator of inflammatory processes (45).

Recent advances in engineered microbiomes and FMT have shown promise in modulating the immune environment and improving therapeutic outcomes, opening new avenues for personalized and precision medicine (46, 47). The modulation of the gut microbiota and bile acid profiles has emerged as a promising therapeutic strategy for gastrointestinal disorders, underscoring the significance of elucidating the complex dynamics within the bile acid–microbiota axis (48).

Previous studies (49, 50) have shown that *Eubacterium fissicatena*, a microorganism closely linked to inflammatory conditions, is strongly associated with obesity-related metabolic disorders. Its abundance level positively correlates with the severity of non-alcoholic fatty liver disease. Additionally, Misiak et al. (51) found that *Eubacterium fissicatena* may be involved in activating immunoinflammatory processes, such as altering intestinal barrier permeability, interfering with lipid metabolism, and disrupting glucose homeostasis. Therefore, *Eubacterium fissicatena* is considered a potential disease-associated bacterium that may play an unfavorable role in the development of various diseases (52). In our study, *Eubacterium fissicatena* was the key bacterial group identified by MR-BMA analysis as affecting the proportion of Effector Memory CD8^+^ T cells among total T cells, thereby inducing the development of nasal polyps.

*Prevotellaceae* are dominant commensal gut microbiota that play a crucial role in regulating host immunity and metabolism by producing short-chain fatty acids (e.g., butyrate) (53, 54). Ruiz-Saavedra et al. (55) found that the abundance of *Prevotellaceae* was significantly higher in healthy controls than in patients with intestinal polyps and adenomas. Notably, the abundance was even lower in patients with adenomas compared to those with intestinal polyps. This finding suggests that *Prevotellaceae* play a crucial role in maintaining intestinal homeostasis and preventing intestinal diseases. Our study suggests that *Prevotellaceae* protect against nasal polyps, potentially by reducing their risk through inhibiting CD39 levels on CD39^+^ activated CD4 regulatory T cells and CD45RA on naive CD4^+^ T cells.

*Deltaproteobacteria*, an important microbial taxon, play a key role in the material cycle of nature, with their metabolic activities being important for the transformation of elements such as carbon, sulfur, and heavy metals (56, 57). However, in humans, *Deltaproteobacteria* are considered pathogenic microorganisms that colonize both the oral cavity and the intestinal tract, and are associated with the development of periodontitis and other systemic diseases (58, 59). Terminally differentiated CD8^+^ T cells comprise a wide range of cell types, with cytotoxic T lymphocytes (CTLs) being the predominant and most typical type (60). Studies (61) have shown that the nasal cavity is a unique immune site where CTLs play an important role in the local immune defense mechanism and in the pathogenesis of atypical allergic rhinitis (NARES) and chronic nasal mucosal inflammation (62). It is noteworthy that there is a large CTL infiltration in the nasal mucosal tissues of NARES patients, and the cytokines and cytotoxic molecules secreted by CTLs can induce mucosal damage, thereby triggering or exacerbating the symptoms of rhinitis. Our study found that both *Deltaproteobacteria* and terminally differentiated CD8^+^ T cells are risk factors for the development of nasal polyps, and that *Deltaproteobacteria* and terminally differentiated CD8^+^ T cells are also causally associated with each other.

Previous studies (63) have found a significant association between the presence of *Methanobrevibacter*, a methanogenic bacteria taxon, and the incidence of colon polyps, with a higher percentage of these strains detected in patients with colon polyps. In recent years, methanogenic bacteria, besides being intestinal residents, have been found to potentially act as pathogens in diseased tissues, such as muscle and brain abscesses (64, 65). Notably, Sogodogo et al. (66) showed that *Methanobrevibacter* may be involved in the pathogenesis of refractory sinusitis by persistently colonizing the nasal cavity of patients and triggering a chronic inflammatory response. Ali et al. (67) integrated multi-omics data (peripheral blood, feces, and liver tissues), found a reduced abundance of *Methanobrevibacter* in patients with hepatitis C compared to healthy controls, which was negatively correlated with disease severity. However, a systematic review (68) suggested that *Methanobrevibacter* abundance is lower in the gut of obese individuals. *Methanobrevibacter* may play a beneficial role in the intestine by participating in physiological processes such as metabolism and immune regulation, but it may become a potential pathogen and cause disease upon colonizing extraintestinal tissues. This phenomenon highlights the importance of the ecological location of microbial communities in determining their roles. Our study suggests that *Methanobrevibacter* may contribute to an increased risk of nasal polyps by affecting the levels of CCR2 on CD62L^+^ myeloid dendritic cells and CD3 on CD4 regulatory T cells.

As one of the dominant gut genera, *Ruminococcaceae* plays a crucial role in maintaining the diversity and homeostasis of the gut microbial community. However, the abundance of this genus was found to be significantly reduced in the gut of patients with dark spot polyp syndrome, colorectal cancer, and its precancerous lesions (69, 70). Some members of the *Ruminococcaceae* are capable of producing bile acid-stimulating metabolites and are therefore often regarded as beneficial strains (71). However, it is noteworthy that *Ruminococcaceae* is a large taxon containing numerous species, and therefore, individual roles may vary significantly. Jiang et al. (72) showed that *RuminococcaceaeUCG002* may be a harmful strain, with its elevated abundance being associated with a high risk of chronic insomnia and cardio-metabolic diseases. Radjabzadeh et al. (73) further found that *RuminococcaceaeUCG002* was significantly associated with depressive symptoms through double-cohort validation and may play a key role in the pathogenesis of depression. A systematic review suggested that Eubacterium brachy may be a beneficial oral strain, with its higher abundance being associated with a lower risk of periodontal disease (74). Rezasoltani et al. (75) found that salivary Eubacterium brachy may serve as a novel biomarker for colorectal cancer screening. In our study, we found that *RuminococcaceaeUCG002* is a risk factor for colon polyps, with its mechanism of action potentially related to the expression of CD24 on IgD-CD38- B cell subsets. Additionally, Eubacterium brachy was a protective factor against colon polyps, with its mechanism of action being related to CD4 on CD28^+^ CD4^+^ T cells.

*Actinobacteria* are usually considered beneficial gut microbial taxa and play an important role in colorectal tumorigenesis (76). This is consistent with our results that *Actinobacteria* are beneficial microbiota in colon polyps, which showed the highest posterior probability of 0.445 based on MR-BMA. However, for the development of gastric polyps, *Actinobacteria* may be present as pathogens. Alarcón et al. (77) suggest that *Actinobacteria* may be involved in *H. pylori*-associated intestinal metaplasia and gastric carcinogenesis. The reason for this is the interesting finding from previous studies that *H. pylori*-associated gastritis led to a reduction in the diversity of gastric microorganisms, but when gastric microorganisms were present only in *H. pylori* infection, it instead significantly prolonged the emergence of gastric malignant tumors (78). This result was verified by the study of Lertpiriyapong et al. (79), in which even a limited variety of intestinal microbiota could contribute to the *H. pylori*-induced gastric tumorigenesis. This reveals a potential mechanism for the role of gut microbiota in gastric carcinogenesis. We found that *Actinobacteria* could promote gastric polypogenesis by inhibiting the protective effect of PD-L1 on CD14^+^CD16^+^ monocytes. It can also induce gastric polypogenesis by elevating the ratio of CD28^+^ CD45RA^+^ CD8dim T cells to CD8dim T cells.

We identified CD45 on CD66b^++^ myeloid cells as a risk factor for gastric polyps, a class of myeloid-derived cells that highly express CD66b and CD45. Mao et al. (80) found that myeloid-derived suppressor cells (MDSCs) with the CD45^+^CD33lowCD11bdim phenotype depleted L-arginine in the microenvironment through the IL-6/IL-8-arginase I axis and inhibited tumor cell killing by CD8^+^ T cells, and it is noteworthy that this type of MDSCs exhibits concomitant high expression of CD66b. Similar results were obtained by later studies, in which higher numbers of CD66b^+^ neutrophils infiltrating within the tumor tissues of patients with gastric adenocarcinoma and esophageal adenocarcinoma were associated with poorer disease-free survival and overall survival, and this association of high infiltration with poor prognosis was more pronounced in female patients. *In vitro* experiments showed that CD66b^+^ neutrophils could promote the migration and invasive ability of gastric cancer cell lines by secreting inducible epithelial-mesenchymal transition-associated factors (e.g., TGF-β and IL-6). Additionally, we identified CD20 on IgD-CD27- B cells as a protective factor against gastric polyps. CD20 is a cell surface protein predominantly expressed on B cells and is widely used as a marker for identifying and characterizing B cells. IgD-CD27- B cells, also known as double-negative B cells, are a subpopulation of poorly differentiated or naïve B cells characterized by the lack of antigenic experience and memory cell markers. Previous studies (81) have shown that high levels of CD20^+^ B-cell follicles are highly correlated with the prognosis of gastric cancer, and that the level of tumor-infiltrating CD20^+^ IgD-CD27- B cells is positively correlated with the overall and disease-free survival of patients with gastric cancer, who have a better survival prognosis when exhibiting high levels of CD20^+^ IgD-CD27- B-cell infiltration.

Huang et al. (82) constructed a model mouse for chronic atrophic gastritis and analyzed it by high-throughput sequencing, finding that the abundance of *Gastranaerophilales* was significantly reduced in the intestines of the model mouse with chronic atrophic gastritis. By monitoring the dynamics of the gut microbiota in mice, Pessoa et al. (83) found that the abundance of *Gastranaerophilales* was decreased in the intestines of mice fed a high-fat diet. In most studies (84, 85), *Gastranaerophilales* have been recognized as a group of beneficial bacteria with important roles in lipid metabolism *in vivo* and immunity. Terminally differentiated CD8^+^ T cells are characterized by high cell surface expression of CD8. These cells usually undergo multiple rounds of cytokinesis and differentiate into effector cells capable of rapidly producing cytotoxic molecules, such as perforin and granzymes, to destroy target cells (86). During the early stages of *H. pylori* infection, CD8^+^ T cells of the tissue-resident memory phenotype are specific for CagA, the virulence factor of *H. pylori*, and are capable of recognizing the CagA antigen and producing the cytokine IFN-γ, which accelerates *H. pylori* clearance and attenuates gastric mucosal damage and inflammatory responses induced by CagA (87). In our study, both *Gastranaerophilales* and CD8 on terminally differentiated CD8^+^ T cells were protective factors against gastric polyps, and *Gastranaerophilales* also achieved this protective effect through CD8 on terminally differentiated CD8^+^ T cells, realizing this causal pathway.

This investigation presents several strengths. Mendelian randomization analyses were employed to elucidate potential causal relationships between gut microbiota, immune traits, and polyp development, while mitigating the influence of confounding factors. The genetic instruments for gut microbiota were derived from the largest genome-wide association study (GWAS) meta-analysis to date, enhancing the robustness of our MR analytical approach. The inclusion of 731 immune traits ensured comprehensive coverage of the immunophenotypes. To address potential violations of MR assumptions, horizontal pleiotropy and heterogeneity was assessed using MR-PRESSO method and MR-Egger regression intercept test. A two-sample MR design was implemented, utilizing non-overlapping exposure and outcome datasets at the aggregate level to minimize bias.

Notwithstanding these strengths, several limitations warrant consideration. Primarily, the study population was restricted to individuals of European ancestry, necessitating cautious extrapolation of findings to other ethnic groups due to potential genetic heterogeneity. Secondly, constraints in the available datasets precluded exploration of causal relationships between gut microbiota and polyps at the species level. Lastly, the utilization of summary statistics rather than individual-level data precluded subgroup analyses, such as differentiation between hyperplastic polyps and adenomas. These limitations underscore the need for future studies incorporating diverse populations, species-level microbial data, and individual-level phenotypic information to further elucidate the complex interplay between gut microbiota, immune function, and polyp formation.

## 5 Conclusion

Mendelian randomization analysis revealed new evidence for the pathogenesis of polyps, highlighting the crucial roles of gut microbiota and immune status in polyp development. We emphasize the key role that specific gut microbiota and immune traits play in influencing the risk of different types of polyps, including nasal polyps, gallbladder polyps, colon polyps, and gastric polyps. The identification of causal pathways through which gut microbes modulate immune function and thus influence polyp development contributes to a better understanding of the underlying mechanisms. MR-BMA approach prioritizes the importance of specific factors. These findings have potential implications for polyp prevention strategies, early screening efforts, and the reduction of polyp risk through the development of effective interventions targeting the identified etiologies. Overall, we emphasize the importance of the findings in elucidating the complex interactions between gut microbiota, immune traits, and polyp development, as well as the potential translational application of these insights in clinical practice and disease prevention.

## Data Availability

The original contributions presented in the study are included in the article/**Supplementary Material**. Further inquiries can be directed to the corresponding author.

## Supplementary material

Supplementary File S1 Detailed information on instrumental variables for gut microbiota.

Supplementary File S2 Detailed information on instrumental variables for immune traits.

Supplementary File S3 MR-BMA analysis of gut Microbiota and immune traits.

Supplementary File S4 Mediated MR analysis of gut Microbiota and immune traits.
